# FTY720 in immuno-regenerative and wound healing technologies for muscle, epithelial and bone regeneration

**DOI:** 10.3389/fphys.2023.1148932

**Published:** 2023-05-12

**Authors:** Monica Behara, Steven Goudy

**Affiliations:** ^1^ Wallace H. Coulter Department of Biomedical Engineering, Georgia Institute of Technology, Atlanta, GA, United States; ^2^ Department of Otolaryngology, Emory University, Atlanta, GA, United States

**Keywords:** regenerative medicine, tissue engineering, immunomodulation, FTY720, multiple sclerosis, wound healing, regenerative micro-environment

## Abstract

In 2010, the FDA approved the administration of FTY720, S1P lipid mediator, as a therapy to treat relapsing forms of multiple sclerosis. FTY720 was found to sequester pro-inflammatory lymphocytes within the lymph node, preventing them from causing injury to the central nervous system due to inflammation. Studies harnessing the anti-inflammatory properties of FTY720 as a pro-regenerative strategy in wound healing of muscle, bone and mucosal injuries are currently being performed. This in-depth review discusses the current regenerative impact of FTY720 due to its anti-inflammatory effect stratified into an assessment of wound regeneration in the muscular, skeletal, and epithelial systems. The regenerative effect of FTY720 *in vivo* was characterized in three animal models, with differing delivery mechanisms emerging in the last 20 years. In these studies, local delivery of FTY720 was found to increase pro-regenerative immune cell phenotypes (neutrophils, macrophages, monocytes), vascularization, cell proliferation and collagen deposition. Delivery of FTY720 to a localized wound environment demonstrated increased bone, muscle, and mucosal regeneration through changes in gene and cytokine production primarily by controlling the local immune cell phenotypes. These changes in gene and cytokine production reduced the inflammatory component of wound healing and increased the migration of pro-regenerative cells (neutrophils and macrophages) to the wound site. The application of FTY720 delivery using a biomaterial has demonstrated the ability of local delivery of FTY720 to promote local wound healing leveraging an immunomodulatory mechanism.

## Graphical Abstract

**Figure:**
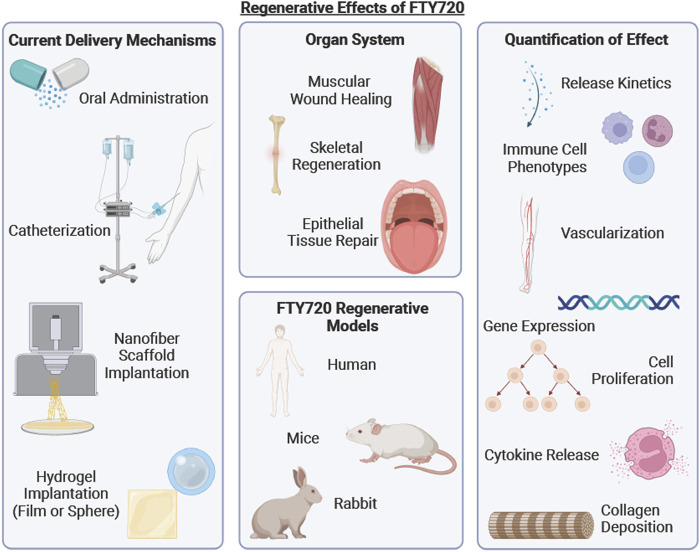
A schematic summarizing the current delivery mechanisms of FTY720, organ systems and regenerative models reviewed and hall marks of wound healing upon FTY720 delivery.

## 1 Introduction

The timeline of normal wound healing consists of 4 distinct phases: hemostasis, inflammation, proliferation, and wound remodeling (Velnar et al., 2009). These phases can be characterized by the influx of specific cell phenotypes which influence the necessary steps of tissue regeneration including vascularization, cell proliferation/migration and collagen deposition. Immediately following injury, the hemostasis and coagulation phase recruits platelets to the wound edge, leading to the formation of a hemostatic plug to stop bleeding (Ellis et al., 2018). The degranulation of the platelets releases pro-inflammatory cytokines such as IL-8 and TNF- α that indicate the beginning of the inflammatory stage of wound healing. Platelets also promote angiogenesis in resident endothelial cells through the secretion of VEGF (Velnar et al., 2009). During the inflammatory stage, neutrophils are recruited to remove debris and they release key cytokines that then recruit monocytes 5-6 h after the onset of the inflammatory stage (Temenoff and Mikos, 2008). Macrophages are also recruited to the site of injury during the inflammatory stage, but generally arrive 8 h after injury becoming the predominant cell type over the ensuing days and weeks (Temenoff and Mikos, 2008). Macrophages secrete cytokines that activate fibroblasts, keratinocytes and endothelial cells via chemical mediators such as tissue growth factors like TGF- β, collagenase and fibroblast growth factor (Velnar et al., 2009). At the end of the inflammatory phase, lymphocytes are attracted to the site of injury, producing cytokines that aid in collagen deposition and tissue remodeling (Temenoff and Mikos, 2008; Velnar et al., 2009). Prolongation of the inflammatory phase can prevent the proliferative phase from initiating, leading to poor wound healing. Following the inflammatory phase, the body transitions into the proliferation phase, where fibroblasts deposit extracellular matrix components that support cell migration and collagen deposition. The hypoxic condition of the wound during the inflammatory stage leads to the production of VEGF which promotes angiogenesis, via mitosis of endothelial cells (Velnar et al., 2009). The final phase of wound healing is remodeling, which involves the formation of new epithelium as well as maturation of the collagen deposition into scar tissue formation, that may last up to 1 year post injury (Velnar et al., 2009). This final recovery step of the remodeling phase involves the alignment of collagen fibers, and reduced macrophage and monocyte populations at the site of injury, supplemented by decreased metabolic activity, concluding the cycle of wound healing (Velnar et al., 2009).

Aberrant wound healing occurs due to prolongation of one of the phases of wound healing, leading to clinical problems like volumetric muscle loss, venous stasis ulcers or hypertrophic scarring, due to increased immune cell populations at the site of injury. Failure to control the body’s innate immune response to injury leads to aberrant wound healing and significant morbidity. Therefore, a pressing need for a therapy to harness the innate immune system is needed. The ability to harness the innate immune system will control the inflammatory component of wound healing by limiting lymphocyte egress, decreasing the pro-inflammatory immune cells that prevent the proliferative phase from initiating (Guo and DiPietro, 2010). FTY720, also known as Fingolimod, is currently being investigated as an immunoregenerative therapy to stimulate skeletal, epithelial, and muscular regeneration by controlling the innate immune responses using *in vivo* wound healing models. FTY720 is currently FDA approved to treat relapsing multiple sclerosis, which potentially shortens the route for repurposing the use of FTY720 in wound healing, leveraging its immunoregenerative effects (Podbielska et al., 2013).

This current in-depth review focuses on the regenerative effects of FTY720 when delivered following muscle, bone, and epithelial injury. We will discuss the current biomaterial therapeutic delivery options as well as the potential of FTY720 to be applied in a variety of other regenerative use cases. To evaluate the potential applications of FTY720, each article was analyzed for defect type, biomaterial treatments, injury location and animal model to determine the effectiveness of FTY720 on wound healing in that defect. Release kinetics of the biomaterial treatments, and the regenerative effects, including collagen deposition, vascularization and immune cell recruitment were then assessed for comparison to current standards of wound healing. To conclude this review, an assessment of these key factors was compared to determine the effectiveness of FTY720 as an immunotherapy for wound healing.

## 2 Mechanism of action of FTY720

FTY720, or Fingolimod, is a Sphingosine-1-Phosphate (S1P) lipid mediator that has been proven to be effective in reducing lymphocyte egress from lymph nodes to stop lymphocyte infiltration into the efferent lymph, which is used to treat relapsing multiple sclerosis (Baer et al., 2018). S1P represents a class of sphingolipids that are produced by the phosphorylation of sphingosine (Bryan and Del Poeta, 2018). S1P and its related pathways are essential to cell proliferation, apoptosis, and angiogenesis as it is a naturally occurring sphingolipid that exists in a variety of tissues (An et al., 2000; Bryan and Del Poeta, 2018). To identify members of the S1P sphingolipid class, cells can exhibit one of five main S1P G-coupled receptors. S1P receptors 1–3 (S1P_1-3_-Rs) specifically control lymphocyte egress and are expressed on a variety of cells, such as immune and cardiovascular cells (Urbano et al., 2013). S1P_4_-R and S1P_5_-R are specifically located on lymphatic tissues and the white matter in the central nervous system respectively (Wang et al., 2019).

### 2.1 Phosphorylation of FTY720

Phosphorylation of FTY720 allows it to become bioactive in humans as well as animal models (Billich et al., 2003). Once FTY720 has been phosphorylated, it then becomes available to bind to the externalized G-coupled protein receptors on lymphocytes entering the node. FTY720 is phosphorylated through sphingosine kinases, which are a class of enzymes that catalyze the formation of S1P (Billich et al., 2003). Given that FTY720 is a functional antagonist of S1P, sphingosine kinases are effective in phosphorylating FTY720 to its bioactive form FTY720P (Paugh et al., 2003). Specifically, FTY720 is phosphorylated via SPHK1 and SPHK2 which occurs primarily in the liver (Qi et al., 2019). However, there have been several studies that have depicted that SPHK1 is present in the lung and spleen tissue and SPHK2 is present in heart and liver (Wadgaonkar et al., 2009). Additionally, murine studies show that aside from the lung and spleen, SPHK1 activity was next highest the brain, kidney, and lymph nodes (Billich et al., 2003). The presence of SPHK2 was also noted in this murine study, reporting that the presence is highest in the liver, kidney, brain and lung (Billich et al., 2003). It has been shown that due to limitations in K_m_ of SPHK1, SPHK2 is much more efficient in phosphorylating FTY720, and mainly occurs in the liver (Paugh et al., 2003; Qi et al., 2019). It is also important to note that with *in vitro* studies, the bioactive form, or FTY720-P must be introduced to cells, especially monocyte and macrophage populations as the drug is phosphorylated *in vivo* (Mullershausen et al., 2009). The phosphorylation mechanism cannot be carried out in macrophages as macrophages do not have the presence of SPHK1 or SPHK2. Once phosphorylated, FTY720 can enter the lymph node to bind to the lymphocyte S1P_1_ receptors.

### 2.2 Mechanism of action of FTY720 in the lymph node

**FIGURE 1:**
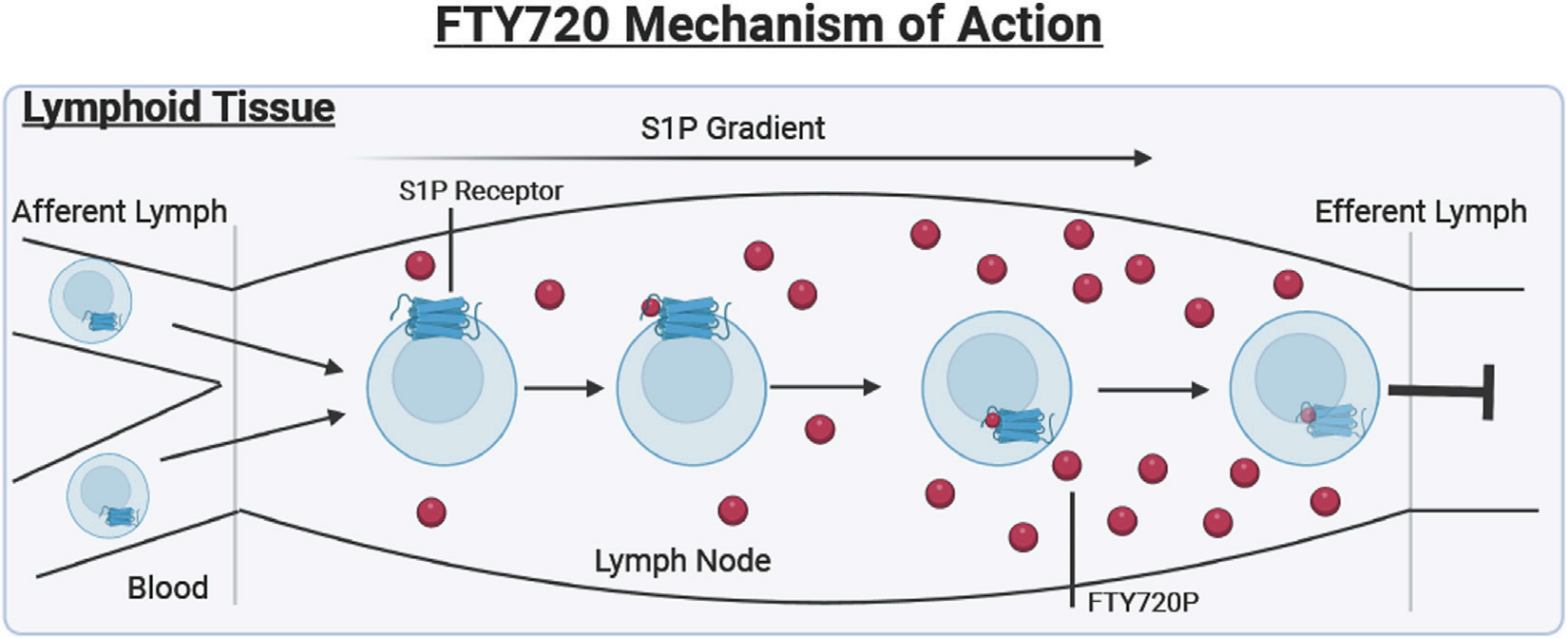
Lymphoid tissue. Describes the mechanism of action of FTY720 (Shown in red). Lymphocytes enter the lymph node from the blood and afferent lymph. The S1P receptor is externalized on the cell surface where it binds to the bioactive FTY720P resulting in the internalization and degradation of the receptor, preventing lymphocyte egress into the efferent lymph.

The drug FTY720 targets S1P_1_- Rs for internalization and degradation to deter lymphocyte egress from the lymph nodes and thymus (Brinkmann et al., 2004; Baer et al., 2018). S1P_1_- Rs are expressed on several types of immune cells, but FTY720 specifically works on lymphocytes that are entering the lymph node via afferent lymph, as shown in Figure 1.

During a normal immune response, when lymphocytes enter the lymph node, their S1P_1_- Rs are initially internalized. The main purpose of these S1P_1_- Rs is to guide the cell through lymph nodes that contain a gradient of S1P not found in other tissues (Martínez-Morales et al., 2018). Therefore, once these lymphocytes enter the lymph node, the S1P receptor is externalized. The increased concentration of S1P inside the lymph node results in the desensitization of the receptor, which would then enable the lymphocytes to travel from the lymph node into the tissues, where the S1P concentration is also relatively low (Martínez-Morales et al., 2018).

FTY720 has a very similar chemical structure to S1P which allows the drug to be used as an S1P antagonist, binding to already externalized S1P_1_-R inside the lymph node (Chun and Hartung, 2010). The internalization and eventual degradation of the S1P receptor due to FTY720P binding the S1P_1_-R prevents the egress of lymphocytes into the efferent lymph reducing the number of inflammatory cells in the wound (Brinkmann et al., 2004). This process is further visualized in Figure 1, where the FTY720 is shown to be binding to the G-coupled protein S1P_1_ receptor.

## 3 Delivery mechanisms of FTY720

### 3.1 Current therapeutic deliveries

In 2010, the FDA approved the oral administration of FTY720, as a therapeutic to treat relapsing forms of Multiple Sclerosis (MS), a debilitating auto-immune disease (Chun and Brinkmann, 2011). Multiple sclerosis (MS) is a primary target for the administration of FTY720 due to the central role of lymphocytes in central nervous system injury in MS patients. A pathological hallmark of multiple sclerosis is the loss of the myelin sheath in the central nervous system (CNS), due to the infiltration of auto-regressive lymphocytes across the blood brain barrier. These auto-regressive lymphocytes release pro-inflammatory cytokines IFN-γ and IL-10 which lead to the degradation of the myelin sheath (Schmitz and Chew, 2008). Despite the body’s ability to repair the myelin sheath after injury, the consistent inflammatory attacks from the auto-regressive lymphocytes cause the death of oligodendrocytes rendering the repair mechanisms ineffective (Ghasemi et al., 2017). One of the primary functions of FTY720 is the sequestration of lymphocytes within lymph nodes and thymus, which has been found to protect the CNS from the demyelination of the axons in MS. The sequestration of lymphocytes in secondary lymphoid organs (lymph nodes and thymus) occurs through an interaction with G coupled protein receptors, effectively reducing the presence of lymphocytes in the blood stream and the brain. This interaction with G coupled protein receptors involve FTY720 binding to the S1P G-coupled protein receptor, causing the internalization and eventual degradation of the S1P receptor, preventing lymphocyte egress, as described in Figure 1 above (Chun and Hartung, 2010). This prevents the lymphocytes from entering circulation, so the pro-inflammatory cytokines are therefore not released in the brain, preserving the myelin sheath. FTY720 is typically orally administered to patients with relapsing MS at a dosage of 0.5 mg/day, which is also the FDA approved dosage level for FTY720 (Sharma et al., 2011; Yeh and Weinstock-Guttman, 2011). Dose limiting adverse effects of higher FTY720 doses occur including headache, diarrhea, and nausea. Efficacy of FTY720 to treat relapsing MS was demonstrated by several clinical trials showing that the prevalence of lesions in the brain via magnetic resonance imaging (MRI) scans decreased, denoting slowing signs of relapse (Kappos et al., 2006). In clinical trials, relapse symptoms such as ataxia, and paroxysmal symptoms are assessed by a neurologist, with an MRI scan to corroborate their conclusions (Avasarala, 2017). The MRI scan is assessed for lesions and blood-brain barrier integrity (Avasarala, 2017). In one clinical trial, it was found that MS patients who received FTY720 treatment had a median of 1–3 lesions after 6 months, whereas the placebo group had a median of 5 lesions after the same amount of time (Kappos et al., 2006). In this study, the treatment groups were split into oral administration of FTY720 at 1.25 mg or 5 mg, or a matching placebo, once daily and were assessed for signs of relapse after 6 months. Relapse rates in groups treated with FTY720 were only 35%, as compared to placebo at 77% (Kappos et al., 2006).

An important consideration in the use of FTY720 to treat relapsing MS is the immunosuppressive effect due to the sequestration of CD4^+^ lymphocytes in the lymph nodes, and other immune cell functions. CD4^+^ lymphocytes are crucial in activating cells of the adaptive and innate immune system, mobilizing macrophages and B-lymphocytes (Luckheeram et al., 2012). Due to the important role that the CD4^+^ lymphocytes play in immunity, patients taking FTY720 are more at risk for infection due to their relative immunocompromised state. (Hirahara and Nakayama, 2016). Upon treatment with FTY720, it has been shown that the sequestration of CD4^+^ lymphocyte within the lymph nodes will not cause lymph node enlargement, as only 2% of the lymphocytes are meant to be in the peripheral lymph at any given time (Chun and Hartung, 2010; Mazzola et al., 2015). Additionally, FTY720 has not been found to have an effect on T and B cell function, which suggests that the harmful naïve T cells that are releasing cytokines that degrade the myelin sheath upon constant attack are being sequestered (Chun and Hartung, 2010).

### 3.2 *In vivo* study delivery mechanisms

#### 3.2.1 Polymer based scaffolding

There are several types of polymer based scaffolding for wound healing and drug delivery purposes, based on the tissue type of the injury. Nanofiber scaffold implantation is a common methodology for drug administration. This process involves the reconstitution of the drug into a polymer solution which is then spun into nanofibers to create a scaffold that can be implanted. This common type of scaffolding usually consists of a combination of poly (lactic-co-glycolic acid) (PLGA) and polycaprolactone (PCL) along with the drug that can be diluted in solvent (Nemati et al., 2019). Once the solution is made, scaffolds are created using electrospinning technology (Xie et al., 2020). Electrospinning is a technique where a high voltage is applied to a polymer and drug solution of certain viscosity, resulting in the outward spinning of fibers via the Taylor’s cone (Xie et al., 2020). The spherical droplet of the viscous solution is deformed into a conical shape opposing surface tension as it is pushed through the metallic needle which then collects on a collecting plate some distance from the tip of the needle (Haider et al., 2020).

Though nanofiber scaffolds are quite common, this review also examines studies that utilize microspheres, thin films and polymer coated allografts to delivery FTY720. In these articles, the microsphere biomaterial is made via the single emulsion method, where the PLGA and drug are dissolved via sonication and the solution is then slowly ejected into Poly vinyl alcohol while stirring overnight (Das et al., 2014b). Microsphere fabrication will result in different release kinetics, altering the overall effect and release of FTY720 compared to the nanofiber delivery system. Thin films were also implanted in muscle injury defects. These films are formed by mixing a polymer, such as PLGA, with a solvent, such as dichloromethane. After introducing a drug into the solution, the liquid is casted and dried at −20°C for 7 days (San Emeterio et al., 2017). The papers reviewed here are do not characterize release kinetics from these thin films but demonstrate efficacious results in their respective injury model. Lastly, Huang et al., 2012, also characterized FTY720 release from a drug/polymer coated allograft. Here the bone allograft was vortexed with a solution of PLAGA, FTY720 and dichloromethane for 24 h, to form a coating (Huang et al., 2012). The various polymer based scaffolding techniques were analyzed for release kinetics of FTY720, and showed varying effects on wound healing, with regard to factors such as collagen deposition, vascularization and immune cell recruitment.

##### 3.2.1.1 Polymer based scaffolding fabrication methodology and release kinetics

In this review, several biomaterial treatments for FTY720 administration are described, including nanofiber and microsphere delivery. San Emeterio et al., 2021, Amanso et al., 2021, and Ballestas et al., 2019 all delivered FTY720-loaded PLGA/PCL nanofibers to implant into mice (Ballestas et al., 2019; Amanso et al., 2021; San Emeterio et al., 2021). Das et al., 2014b, on the other hand, utilized microspheres as a method of implantation (Das et al., 2014b).

The release kinetics of FTY720 from a scaffold will determine the size of scaffold for implantation, dosage of FTY720 for the animal and will allow the quantification of drug release to correlate with the effects of tissue regeneration. In nanofiber scaffold form, the exact concentration of drug and release time are evaluated via UV-Vis spectroscopy, Liquid Chromatography Mass Spectrometry (LCMS) and High Performance Liquid Chromatography (HPLC) (Das et al., 2014b; San Emeterio et al., 2021; Yang et al., 2022). Using these evaluation methods, it has been demonstrated that FTY720 scaffolds generate a burst release that typically occurs within the first 24 h of implantation, with minimal release on the following days, as quantified by a percentage release overall. This was demonstrated by San Emeterio et al., 2021, where the FTY720-nanofiber released 96% of FTY720 in the first 24 h, as well as by Yang et al., 2022, where a burst release of 37.95% of FTY720 from the nanofiber was noted on the first day (San Emeterio et al., 2021; Yang et al., 2022). FTY720 release from microsphere demonstrated a burst release using HPLC by Das et al., 2014b, where 25% of the FTY720 was released in the first 20 min (Das et al., 2014b). Long term release from a nanofiber microsphere was also identified and assessed by Das et al., 2014a, where it was found that 70% of the FTY720 was released over a total of 4 weeks (Das et al., 2014a). Additionally, Li et al., 2019, Amanso et al., 2021, Ballestas et al., 2019 all reported a long term release of 85%–100% (Ballestas et al., 2019; Li et al., 2019; Amanso et al., 2021). There were some papers, such as Awojoodu et al., 2013, that also utilized PLAGA/PCL nanofiber scaffolds contain FTY720, but did not characterize the release kinetics, but more so the regenerative effects of the FTY720 in a muscle model (Awojoodu et al., 2013). The other polymer based scaffolding techniques found similar results; for example, the drug/polymer coated allograft developed by Huang et al., 2012 showed 53% release of 5 days (Huang et al., 2012). Though different from nanofiber scaffold release, it must be noted that the allografts have a different method of implantation and therefore will have different regenerative effects. Overall, it was found that the polymer based scaffold biomaterials resulted in significant FTY720 release in both immediate and long acting studies.

Table 1 compares the delivery, animal models, and release kinetics of FTY720.

**TABLE 1:** A review of the delivery mechanisms, animal models, biomaterial and release kinetics of studies pertaining to the administration and delivery of FTY720 into an animal model.

Delivery mechanism	Animal model	Composition	Release kinetics	Citation
Scaffold Implantation	Female Sprague Dawley Rats	FTY720 PLGA Microspheres	Burst: N/A	Das et al. (2014a)
			Long Term: 70% over 4 weeks	
	Sprague Dawley Rats	FTY720 loaded MGB-PLGA nanofiber scaffold	Burst: ∼10% on day 1	Li et al. (2019)
			Long Term: 85% over 8 weeks	
	Rats	FTY720 and BMSC loaded silk fibrin and gelatin 3D printed scaffold	Burst: 37.95% on day 1	Yang et al. (2022)
			Long Term: 66.31% over 2 weeks	
	Male Sprague Dawley Rats	50:50 PLGA Microsphere or 85:15 PLGA Microspheres	Burst: 85:15%–25% of FTY in 20 min; 50:50–N/A	Das et al. (2014b)
			Long Term: N/A	
	C57BL/6 Mice	FTY loaded PLGA/PCL nanofiber scaffold	Burst: 96% on day 1	San Emeterio et al. (2021)
			Long Term: 97% at 75 h	
	C57BL/6 Mice	FTY720 PLGA/PCL nanofiber scaffold	Burst: N/A	Amanso et al. (2021)
	C57BL/6 Mice	FTY720 PLGA/PCL nanofiber scaffold	Long Term: Near 100% release at 80 h	Ballestas et al. (2019)
	C57BL/6 Mice, NG2–DsRed mice, CX3CR1–eGFP mice	FTY720 PLAGA/PCL nanofiber scaffold		Awojoodu et al. (2013)
Drug/Polymer Coated Allograft	Female Sprague Dawley Rats	FTY720 coated bone allograft	Burst: N/A	Huang et al. (2012)
			Long Term: 53% over 5 days	
“Film” Implantation	Male C57BL/6 Mice	FTY720-loaded Dichloromethane film		San Emeterio et al. (2017)
Hydrogel Implantation	Male C57BL/6 mice	Heparin and PEG-DA hydrogel	Burst: N/A	Ogle et al. (2017)
			Long Term: 87.2% in 7 days	

#### 3.2.2 Hydrogel implantation

Hydrogel fabrication involves the formation of a network of polymeric chains that join via physical or chemical crosslinking (Gulrez et al., 2011; Yang et al., 2018). There are several polymers that can be utilized for applications *in vivo*, such as PEG, gelatin, and collagenase, as well as a variety of crosslinking methodologies such as UV light, chemical crosslinking or heat treatment (Delattre et al., 2019). Hydrogels are currently available for clinical use as a gel or sheet, as manufactured by companies such as Smith & Nephew and McKesson (Sivaraj et al., 2021). However, the seeding of stem cells can create a bioactive hydrogel scaffold, that could be beneficial to wound healing in the future (Sivaraj et al., 2021). The novel, key feature of a hydrogel scaffold is its ability to swell and create a 3-dimensional structure due to the hydrophilic polymers that are crosslinked in the biomaterial. Yang et al., 2022 utilized FTY720 in a hydrogel scaffold form, where they used methacrylate silk fibroin and gelatin with bone marrow stem cells seeded into the scaffold to implant into mice (Yang et al., 2022). Overall, the porosity, durability, charge and biocompatibility are all features that can be modified based on the fabrication of the specific hydrogel, as well as the cell type to be seeded in the case of hydrogel scaffolds (El-Sherbiny and Yacoub, 2013). Release of FTY720 from hydrogels show similar release kinetics, though slightly less than that of a scaffold implantation. Ogle et al., 2017 developed a FTY720-hydrogel implantation model and showed an 80% release of FTY720 over 7 days (Ogle et al., 2017).

#### 3.2.3 Subcutaneous catheterization

There are a variety of papers that use an *in vivo* catheterization to inject the drug into the animal model. The main purpose of *in vivo* catheterization for FTY720 delivery was used in cutaneous injury models to ensure that FTY720 is applied at the site of injury, rather than systemically. The dosage of FTY720 was suspended in ethanol or saline, and measured in microliters of dosage, or mg/kg for each animal. The varying amounts of weight-based dosage pertains to the size of defect targeted. Because this delivery mechanism does not consist of a biomaterial fabrication, the drug dosage pilot studies were utilized to determine optimal dosage amounts (Aoki et al., 2020). Aoki et al., 2020 found the optimal dose was found to be 51.6 μg FTY720/kg/day for their mouse model of hypertrophic scarring (Aoki et al., 2020). It can be assumed that Shi et al., 2017 and Ginestal et al., 2019 administered dosages of FTY720 that are clinically relevant to the animal model studied (Shi et al., 2017; Ginestal et al., 2019). Dosages were administered via subcutaneous injection, where the animal is subjected to the dosage of FTY720, either reconstituted in saline, ethanol and then monitored for the remainder of the study. An overview of dosages and animal models can be viewed in Table 2.

**TABLE 2:** A review of the animal models that received sub-cutaneous catheterization as a method of administration for FTY720.

Animal model	FTY720 solution	Dosage	Citation
Male New Zealand Rabbits	Pure FTY720	5 mg/mL	Shi et al. (2017)
C57BL6/J mice	FTY720, Saline, Ethanol	10 μM at 200 μL	Aoki et al. (2020)
Female Sprague-Dawley rats	FTY720, Saline, Ethanol	0.6 mg/mL	Ginestal et al. (2019)

## 4 Wound healing models for FTY720 administration

### 4.1 Epithelial defects

With FTY720 administration via subcutaneous catheterization and nanofiber scaffolding, the majority of epithelial tissue wounds have shown improved wound healing. Though epithelial wound healing follows the typical steps of wound healing (hemostasis, inflammation, proliferation and remodeling), there are several characteristics that are exhibited by this type of wound healing that are unique. For instance, the importance of re-epithelization in these wounds is critical (Cañedo-Dorantes and Cañedo-Ayala, 2019). Keratinocytes flood the defect, eventually differentiating into epithelial cells. These keratinocytes then stimulate the recruitment of fibroblasts and the eventual differentiation into myofibroblasts, furthering the collagen deposition for extracellular matrix remodeling (Cañedo-Dorantes and Cañedo-Ayala, 2019). Aberrant epithelial wound healing can be split into two distinct categories, acute and chronic wounds; where acute wounds are expected to return to the original organized tissue structure after healing, whereas chronic wounds typically consist of a dysfunctional healing process where there is a failure to progress beyond the inflammatory stage of wound healing (Tottoli et al., 2020). Chronic wounds are typically targeted for FTY720 treatments, as these wounds are often related to a prolonged inflammatory phase of wound healing. Within animal models, the main approaches to treating epithelial wounds occurs via subcutaneous catheterization, or PLGA/PCL scaffolding (Shi et al., 2017; Ginestal et al., 2019; Aoki et al., 2020; Amanso et al., 2021). All studies included assessed the amount of collagen deposition, and vascularization of the newly formed epithelial tissue.

For epithelial wound healing, FTY720 has been used as a treatment for hypertrophic scarring, oronasal fistulas, and cutaneous and subcutaneous defects. Hypertrophic scarring refers to the fibrotic condition that can occur after trauma and severe injury to the cutaneous epithelial tissue (Butzelaar et al., 2016). Hypertrophic scarring is characterized by the presence of myoblasts and the over-deposition of collagen and other extracellular matrix components after injury due to prolongation of the proliferative phase of wound healing. In hypertrophic scarring, therapies are being explored to decrease the deposition of collagen and other extracellular matrix components (Rabello et al., 2014). Currently, there are several treatments to manage hypertrophic scarring, such as compression therapy and silicone gel sheets, there is no treatment that is universally effective in reducing the over-production of matrix elements seen in hypertrophic scarring. FTY720 is being explored as a potential treatment to reduce cell viability in hypertrophic scar fibroblasts (Shi et al., 2017). The majority of epithelial wound healing models generate defects that require the formation of scar tissue to heal the wound. For incisional and excisional cutaneous defect models, FTY720 was delivered using scaffolds or was administered through subcutaneous catheterization, in conjunction with saline and ethanol (Ginestal et al., 2019). The application of FTY720 in these defects was to reduce the inflammatory phase of wound healing and stimulate the production of extracellular matrix components to aid in faster overall would healing. In the catheterization study, the efficacy in epithelial wound healing was determined via the comparison of two drugs used to treat multiple sclerosis: FTY720 and Azathioprine. The analysis conducted by Ginestal et al., 2019 highlighted the immuno-modulatory effect of FTY720 and how controlling the inflammatory phase of wound healing can promote cutaneous wound healing (Ginestal et al., 2019). FTY720 delivery via catheterization showed a 92.97% larger wound closure area in excisional cutaneous defects, with full wound healing after 21 days in incisional cutaneous defects (Ginestal et al., 2019).

To test the impact of FTY720 delivery on oral mucosal wound healing an oronasal fistula (ONF) model was created (Ballestas et al., 2019; Amanso et al., 2021). An ONF occurs when there is abnormal communication between the oral and nasal cavity, which, in normal cases, is separated by the hard and soft palate in the mouth (Zielinska-Kazmierska et al., 2021). Two of the studies reviewed in this paper deliver FTY720 into an oronasal fistula via a drug loaded nanofiber scaffold. By assessing the prevalent biomarkers, cell proliferation and vascularization that was exhibited over time, it was shown that FTY720 does foster a pro-regenerative environment that aids in complete epithelial wound healing of the ONF. Specifically, there were noted to be increased epithelial proliferation associated with increased pro-regenerative neutrophils and macrophages by flow cytometry (Amanso et al., 2021).

### 4.2 Muscular defects

There have been a variety of FTY720 loaded biomaterials used to improve wound healing of muscle tissue. Though the typical stages of wound healing are generally followed in muscle regeneration, there are a few hallmarks that can systemically differentiate the muscular wound healing process from that of an epithelial wound. Muscle regeneration typically begins 4-5 days after injury, but requires the recruitment of adult muscle stem cells, specifically satellite cells, as endogenous muscle fibers do not have the ability to divide (Laumonier and Menetrey, 2016). These satellite cells then recruit and differentiate into myoblasts to repair injured muscle fibers. As the stages of wound healing progress, the extracellular matrix is deposited and is infiltrated by fibroblasts, which are critical in converting myoblasts into myofibers (Laumonier and Menetrey, 2016). However, this process can be impacted by the over-deposition of collagen fibers leading to fibrosis (Li and Huard, 2002). Additionally, the chemokine receptor CX3CR1 is specifically utilized to note the types of pro-inflammatory monocyte populations that are recruited to the site of the defect (Dagkalis et al., 2009; Awojoodu et al., 2013). CX3CR1 is expressed in macrophages, monocytes, microglia, and dendritic cells, and has been found to be increased in inflammatory conditions. The presence of CX3CR1 has also been found to stimulate angiogenesis through the activation of pro-angiogenic factors, which has been found to be beneficial in healing of muscle injuries (Liu et al., 2021). Some studies have also found CX3CR1 is important to the regulation of the macrophage phagocytosis function, which can have significant effects on wound healing (Zhao et al., 2016).

The biomaterial treatments used to delivery FTY720 in volumetric muscle loss (VML) defect models include films and scaffolds. VML arises from the injury of muscular tissue which results in an unsuccessful regeneration mechanism. This unsuccessful regeneration can also be characterized by the influx of pro-inflammatory myeloid cells into the area of injury which can exacerbate the injury to the point that regeneration is no longer possible (San Emeterio et al., 2021). This dysfunctional immune response can lead to fibrosis and loss of function over time (Anderson et al., 2019). The use of FTY720 to treat VML has been found to improve muscle regeneration and decrease the immune cell response to the site of muscle injury. The mechanism by which FTY720 improves VML healing is by stopping lymphocyte egress out of the lymph node, which leads to a decrease in myeloid cell influx to the site of injury (San Emeterio et al., 2021). Delivery of FTY720 in muscular wound healing utilizing a ‘film’ biomaterial implanted into a dorsal skinfold window chamber to observe wound healing in real time, as described in Awojoodu et al., 2013, San Emeterio et al., 2017 (Awojoodu et al., 2013; San Emeterio et al., 2017). The dorsal skinfold window chamber directly exposes the muscle to gain access to the section of injured muscle, which can then be monitored (Laschke and Menger, 2016). Monitoring vascularization of the muscle following injury in a dorsal skinfold window chamber model during the third stage of wound healing allows for quantitative assessment (Laschke and Menger, 2016; Schreiter et al., 2017). Following delivery of FTY720, VML models showed increased muscle regeneration and improved vascularization of the muscle tissue compared to controls, identified through quantitative assessments of muscle fibers alignment and endothelial cell proliferation (San Emeterio et al., 2021). Increased muscle regeneration correlated with increased generation of contractile forces, which improve muscle functionality overall (San Emeterio et al., 2021).

### 4.3 Bone defects

The use of FTY720 during bone healing and regeneration with skeletal bone defects has been evaluated in the regeneration of bone in cranial defects. A variety of biomaterials have been utilized in the wound healing of these skeletal defects, such as FTY720 loaded hydrogels and scaffolds (Huang et al., 2012; Das et al., 2014a; Das et al., 2014b; Li et al., 2019; Yang et al., 2022). To study skeletal wound healing, cranial defects allow the researchers to circumvent the need for defect stabilization in a clinically relevant setting (Samsonraj et al., 2017). Given the structure and size of cranial defects, hydrogels and scaffolds can be placed into the defect, and rendered to the correct size. The process of creating a cranial defect utilizes a trephine of proper size that can create a hole in the skull (Samsonraj et al., 2017). This technique is used to simulate cranial injury in all bone wound healing papers assessed in this review. The deposition of bone, angiogenesis and cell recruitment were assessed to show overall osteogenesis with FTY720 treatment (Huang et al., 2012; Das et al., 2014a; Das et al., 2014b; Yang et al., 2018; Yang et al., 2022). Of the 5 studies delivering FTY720 to cranial bone defects, 4 studies demonstrated bone regeneration *in vivo*, with quantifications in osteogenesis, whereas 1 only noted the presence of a pro-osteogenic signaling pathway.

## 5 Common immune cell phenotypes during wound healing

During healing of different types of wounds (epithelial, muscular and bone regeneration) there are populations of pro-regenerative and pro-inflammatory immune cell phenotypes that are commonly present. Progenitor origin of immune cells dictates that the phenotype of each cell is determined from myeloid stem cells, that then differentiate into myeloblasts and then monocytes (Torang et al., 2019). Monocytes can differentiate into dendritic cells and macrophages, which then differentiate into pro-inflammatory (M1) macrophages and anti-inflammatory (M2) macrophages (Torang et al., 2019). These leukocytes can be typically characterized by a surface antigen, CD11b, where positivity indicates the presence of several phenotypes of immune cells, including monocytes, macrophages, granulocytes and natural killer cells (Rhein et al., 2010). When determining specific phenotypes, the CD11b cells are typically used as an preliminary gate during flow cytometry, where positivity indicates the onset of inflammation, subclassifying further with more specific markers (Duan et al., 2016). When discussing monocytes, many papers reviewed here describe the specific surface antigen, Ly6C. Monocytes are a part of the mononuclear phagocyte system, and can be separated into the classical, or pro-inflammatory monocytes, or the non-classical or the pro-regenerative monocyte populations (Mukherjee et al., 2015). A low presence of the Ly6C surface marker indicates a population of anti-inflammatory monocytes, which is favorable for epithelial, muscular, and skeletal wound healing. With regard to macrophages, there are several surface antigens that can be analyzed to distinguish between macrophage phenotypes. The macrophage surface marker CD206 positivity indicates the presence of M2 macrophages, also known as the pro-regenerative macrophage, that are beneficial to wound healing (Xu et al., 2020). In the realm of macrophages, CD68 is another surface antigen that is highly specific to macrophage activation, where the upregulation of this particular marker can indicate a macrophage specific respond to inflammatory stimuli (Chistiakov et al., 2017).

A comparison of FTY720 based tissue regenerative outcomes are shown in Table 3 for epithelial defects, Table 4 for muscular injury models and Table 5 for skeletal models.

**Table 3:** The regenerative effects of FTY720 are assessed by evaluating three key criteria in regard to wound healing: immune cell recruitment, angiogenesis and collagen deposition in epithelial defects.

Biomaterial	Defect type	Immune cell phenotypes *increase = (I), decrease = (D)	Angiogenesis	Collagen deposition	Citation
Catheterization	Abnormal Scarring	-CD206+ (M2) macrophages (I)	—	—	Aoki et al. (2020)
		-M1 macrophages (D)			
		-White Blood Cells (D)			
		-Neutrophils (I)			
	Cutaneous Defect	-M2 macrophages (I)	+	+	Ginestal et al. (2019)
		-Lymphocytes (D)			
		-M1 macrophages (I)			
	Hypertrophic Scarring	-CD68^+^ macrophages (D)		—	Shi et al. (2017)
		-Dermal Fibroblasts (D)			
		-Hypertrophic Scarring Fibroblasts (D)			
Hydrogel	Dorsal Skinfold Window Chamber	-Ly6C low monocytes (I)	+		Ogle et al. (2017)
		-CD11b+Ly6C low (I)			
		-CD68^+^ CD206+ macrophages (I)			
Scaffold	Oronasal Fistula	-CD206lo neutrophils (I)			Amanso et al. (2021)
		-Ly6C low monocytes (I)			
		-M2 Macrophages (I)			
	Oronasal Fistula	-Ly6C low monocytes (I)			Ballestas et al. (2019)
		-M2 macrophages (I)			

**TABLE 4:** The regenerative effects of FTY720 in muscular defects are assessed by evaluating three key criteria in regard to wound healing: immune cell recruitment, angiogenesis and collagen deposition.

Biomaterial	Defect type	Immune cell phenotypes *increase = (I), decrease = (D)	Angiogenesis	Collagen deposition	Citation
Film	Dorsal Skinfold Window Chamber	-CD11b+ inflammatory cells (D)	+		Awojoodu et al. (2013)
		-CD206+ macrophages (I)			
		-CX3CR1+ cells (I)			
		-M2 macrophages (I)			
	Volumetric Muscle Loss	-CD206+ macrophages (I)		+	San Emeterio et al. (2017)
		-CX3CR1 cells (I)			
Scaffold	Volumetric Muscle Loss	-Ly6C^low^ monocytes (I)	+	+	San Emeterio et al. (2021)
		-CD68^+^ macrophages (D)			
		-Satellite Cells			

**TABLE 5:** Skeletal wound healing was assessed, noting the genes that were expressed, cell recruitment, vascularization, and osteogenesis. The characteristics of wound healing in the skeletal system is slightly different than that of the epithelial and muscular systems; the chart headings have been changed to reflect this analysis.

Biomaterial	Defect type	Cell recruitment + gene markers	Angiogenesis	Osteogenesis	Citation
Scaffold	Cranial Defect	mRNA Expression: VEGFA, HIF-1α, RUNX2, OPN, Col1a, OCN		+	Yang et al. (2022)
	Cranial Defect	mRNA Expression: CXCR4, VEGF-A, HIF-1α	+		Li et al. (2019)
	Cranial Defect	mRNA Expression: SDF-1/CXCR4 expression (I) Cell Recruitment: CD90^+^ cells (I), CD29^+^ cells (I), Bone marrow stromal cells (I), Osteoblast recruitment (D)	+	+	Das et al. (2014a)
	Cranial Defect			+	Das et al. (2014b)
	Cranial Defect	Cell Recruitment: Endogenous host stem and progenitor cells (I)	+	+	Huang et al. (2012)

## 6 Discussion

The efficacy of FTY720 on wound healing has been demonstrated by the studies presented and the various drug delivery approaches. The primary modalities of FTY720-associated wound healing were pro-regenerative immune cell recruitment, vascularization, collagen deposition and mRNA marker expression. Though each organ system has a slightly different mechanism of wound healing, these features discussed below provide insight into the efficacy of the drug in regard to epithelial, skeletal and muscular regeneration.

### 6.1 Inflammatory cell prevalence

The inflammatory cell response over the period of wound healing allows for the differentiation of several immune cell phenotypes, with a variety of biomarkers and cytokines to indicate recruitment and progenitor type. Most of these biomarkers used to identify particular inflammatory cells are found by staining for a surface antigen, such as Ly6C and CD206 (Lee et al., 2013; Jaynes et al., 2020; Martínez-Carmona et al., 2021). The positivity or negativity of such antigens on the surface of the cell will indicate the phenotype of the cell in question (pro-inflammatory vs. anti-inflammatory).

In the articles reviewed, the monocyte phenotype Ly6C^low^ anti-inflammatory monocyte population was found to be increased in 5 studies following the delivery of FTY720 treatment (Ogle et al., 2017; San Emeterio et al., 2017; Ballestas et al., 2019; Amanso et al., 2021; San Emeterio et al., 2021). The treatment of FTY720 was also found to increase the recruitment of CD206+ cells in 5 of 9 studies, showing that there is an increase of pro-regenerative or M2 macrophages (Xu et al., 2020). San Emeterio et al., 2017 provided an in-depth analysis of the increase in CD206+ macrophages over a 7-day period, showing that the CD206+ macrophages steadily increased during this time period, corresponding to inflammatory phase of wound healing (San Emeterio et al., 2017). Aoki et al., 2020 and Awojoodu et al., 2013, also showed a decrease in M1 or pro-inflammatory macrophages as well as fewer white blood cells following FTY720 delivery (Awojoodu et al., 2013; Aoki et al., 2020). Shi et al., 2017 shows that the viability of dermal fibroblasts in the case of hypertrophic scarring was reduced (Shi et al., 2017). Ginestal et al., 2019 explored the influx of lymphocytes to the site of injury, in relation to the formulation of granulation tissue (Ginestal et al., 2019). In this study, FTY720 limits lymphocyte egress from the lymph node, but this decrease was not sufficient to fully impair the formation of granulation tissue, which allows the macrophages to become the predominate immune cell phenotype and accelerate wound healing (Ginestal et al., 2019).

In skeletal wound healing, the chemotactic mediators that are released from the periosteum of the injured bone recruit fibroblasts, mesenchymal stem cells and osteoprogenitor cells (Das et al., 2014a; Loi et al., 2016). SDF-1 is one chemokine of interest that has been found to act as a chemotactic for several leukocytes, and is involved in cell migration (D'Apuzzo et al., 1997). As described in Das et al., 2014a, FTY720 enhances the SDF-1 mediated chemotaxis of a certain subtype of bone progenitor cells following bone injury *in vivo* (Das et al., 2014a). Additionally, the transmigration of bone marrow stromal cells and osteoblasts was analyzed, specifically in Das et al., 2014a, demonstrating that FTY720 has a stimulatory effect causing transmigration and greater motility of these cells to the site of injury (Das et al., 2014a). Huang et al., 2012 also suggested that the introduction of FTY720 into this environment encourages the recruitment of endogenous host stem and progenitor cells to the site of injury (Huang et al., 2012). Overall, the immune cell recruitment in epithelial, muscular, and skeletal wound healing can be characterized by distinct cell types that have been found to be stimulated by the treatment of FTY720.

### 6.2 Collagen deposition

Collagen deposition is an important step during the regeneration of injured tissue. The deposition of collagen fibers has been proven to provide support and migration cues for endothelial cells to promote vascularization, and has been widely accepted to be a precursor to the process of vascularization as a whole (Senk and Djonov, 2021). In a normal wound healing cascade, collagen deposition occurs in the proliferation stage, which is characterized by the influx of fibroblasts to the site of injury (Mathew-Steiner et al., 2021). The process of collagen deposition begins with the synthesis of collagen III into the granulation tissue, and is slowly replaced by collagen I over time, signifying the maturation of collagen (Mathew-Steiner et al., 2021). This newly synthesized collagen is further enhanced into a mature collagen matrix via covalent cross-linking, crucial to tissue tensile strength (Mathew-Steiner et al., 2021). Of the 14 papers reviewed, 6 papers studied collagen deposition in epithelial and muscular injury models with FTY720 delivery. Though epithelial and muscular wound healing rely heavily on the deposition of collagen, the majority of the studies did not include a quantitative analysis. However, the studies that did quantify collagen deposition used immunofluorescence and western blots of collagen I, and collagen III to assess the impact of FTY720 (Shi et al., 2017). Aoki et al., 2020 reported collagen deposited by determining the percentage of area that was covered by new collagen (Aoki et al., 2020). Imaging techniques were also used to determine the prevalence of mature and immature collagen as assessed in Ginestal et al., 2019 (Ginestal et al., 2019). One quantitative ratio of determining collagen production is the volume of collagen to desmin ratio. Desmin plays a large role in the intracellular stabilization, and therefore, the relationship between collagen and desmin is important for proper wound healing characterization (Meyer and Lieber, 2012). In normal wound healing, the deposition of collagen fibers and desmin characterizes the transition to the proliferation phase. As discussed in San Emeterio et al., 2017, the volume of collagen to desmin ratio was visualized via two-photon microscopy demonstrating that there was more collagen deposition but a reduced collagen to desmin ratio in the presence of FTY720. This indicated less interstitial fibrosis in a volumetric muscle loss model (San Emeterio et al., 2017). Similarly, San Emeterio et al., 2021 qualitatively found increased collagen deposition following FTY720 delivery, when compared to a blank volumetric muscle loss model (San Emeterio et al., 2021). Ginestal et al., 2019 noted increased mature collagen after FTY720 treatment in an excisional epithelial defect (Ginestal et al., 2019).

In hypertrophic scarring injury models, the effects of FTY720 were found to be beneficial by controlling collagen deposition and suppressing the formation of hypertrophic scars. Because hypertrophic scars are a form of aberrant wound healing, Shi et al., 2017 and Aoki et al., 2020 administered the FTY720 treatment directly via subcutaneous catheterization. Staining for markers of hypertrophic scarring found that collagen I and collagen III were significantly reduced by the treatment of FTY720, which in turn suppressed the formation of hypertrophic scars (Shi et al., 2017). Aoki et al., 2020 found significantly less percentage of wound area suggesting less hypertrophic scarring after FTY720 treatment.

The alignment of the collagen fibers has also been evaluated as a potential target of FTY720 therapy. Several papers such as San Emeterio et al., 2021 and Shi et al., 2017 described the highly aligned collagen orientation following treatment with FTY720 in both hypertrophic scarring as well as VML. This was determined via immunofluorescence and two photon microscopy to conclude that there is a qualitatively higher amount of aligned collagen deposition after FTY720 treatment than that of a non-treated scaffold (San Emeterio et al., 2021). A study of the specific types of collagen were not assessed or reported (San Emeterio et al., 2021). In the case of hypertrophic scarring, Shi et al., 2017 and Aoki et al., 2020, found that the integrated density of collagen compared to the area of the defect was significantly less (Shi et al., 2017; Aoki et al., 2020). In the hypertrophic scarring model, a lower amount of collagen deposition is favorable to the decrease the prevalence of scars, suggesting that FTY720 optimizes the collagen production during wound healing.

### 6.3 Angiogenesis and vascularization

In the wound healing process, angiogenesis and vascularization are a highly dynamic response mechanism, that ensures new blood vessels grow at the site of injury, denoting the transition into the proliferative stage of wound healing. There are several angiogenic cytokines, such as vascular endothelial growth factor (VEGF), transforming growth factor beta (TGF-β) and angiopoietin that are critical in wound angiogenesis (Li et al., 2003). Information from the extracellular matrix is a critical prerequisite of angiogenesis as endothelial cells enter into the extracellular matrix to form networks of branching vasculature (Honnegowda et al., 2015). The angiogenesis involved in wound healing can be separated into 5 distinct steps: initiation, amplification, proliferation, stabilization and suppression (Honnegowda et al., 2015). In each stage, angiogenic cytokines such as VEGF, IL-8, TNF-α are secreted by the macrophages and monocytes present at the site of injury (Honnegowda et al., 2015).

Quantification of the angiogenic impact of the delivery of FTY720 was performed in 5 studies using CD68 or CD31 staining to quantitatively assess the macrophages present and quantify the new vascularization, respectively. The remaining articles utilized Microfil Enhanced micro-CT or immunohistochemistry to assess FTY720 induced angiogenesis (Das et al., 2014a; Ginestal et al., 2019). CD68 is a pan-macrophage antigen and CD31 is an endothelial specific marker (Salva et al., 2014; Zander and Farver, 2016). San Emeterio et al., 2021 found that the muscle tissue in volumetric muscle loss formed distinct, branching vasculature in skeletal muscle in the presence of FTY720, as compared to that of the blank nanofiber scaffold implantation (San Emeterio et al., 2021). Awojoodu et al., 2013 utilized CD68 staining to further characterize the CX3CR1–eGFP + cell recruitment caused by presence of FTY720, to view enhanced growth of lectin-positive capillaries. CX3CR1–eGFP + cells are a specific type of macrophage whose presence promotes sprouting of vasculature in muscle tissue correlating to positive capillary growth (Awojoodu et al., 2013; Burgess et al., 2019). Using CD31 staining, Yang et al., 2022, examined the effect of FTY720 treatment on vascular formation during bone repair, showing significantly higher CD31 in the presence of FTY720 (Yang et al., 2022). The increased vascularization ability due to the presence of FTY720 allowed for greater osteo-induction potential for bone healing (Yang et al., 2022). Several other studies such as Li et al., 2019, Huang et al., 2012, state that the treatment of FTY720 provide an enhanced formation of new vessels, as well as the induction of angiogenesis in skeletal and muscular applications (Huang et al., 2012; Li et al., 2019; Qi et al., 2022).

Additionally, in the context of skeletal wound healing, many of studies provide an in-depth analysis of the protein and mRNA expression related to angiogenesis following FTY720 delivery. Both Yang et al., 2022 and Li et al., 2019 described the upregulation of VEGF-A and elevated HIF-1α after administration of FTY720. VEGF-A expression is correlated with new blood vessel growth and represents a critical step in physiological angiogenesis (Ferrara et al., 2003; Li et al., 2019; Yang et al., 2022). The mRNA expression of VEGF-A and HIF-1α in human umbilical vein endothelial cells (HUVECs) were noted in both Yang et al., 2022; Li et al., 2019 as a marker for angiogenesis, *in vitro*, and was measured via real time quantitative PCR (RT-qPCR) (Li et al., 2019; Amanso et al., 2021; Yang et al., 2022). Li et al., 2019 qualitatively described the upregulation of VEGF-A following FTY720 treatment by indicating that there was a 3.4-fold upregulation of this mRNA expression, furthering the conclusion that mRNA expression of VEGF-A is supplemented by the presence of FTY720 (Li et al., 2019). Several papers also discuss the mRNA expression of CXCR4, which, upon binding, can trigger a variety of signaling pathways that are related to cell migration, and hematopoiesis (Bianchi and Mezzapelle, 2020). This marker is explicitly quantified via RT-qPCR analysis conducted in Li et al., 2019, finding that there is a 1.6 fold greater expression level in the presence of FTY720, showing the stimulatory effect on cell migration by FTY720 (Li et al., 2019).

During skeletal wound healing, several papers also described the induction of tubule formation in HUVECs after the administration of FTY720 *in vitro* as discussed in Li et al., 2019, or within a scaffold, as shown in Yang et al., 2022 (Li et al., 2019; Yang et al., 2022). HUVECs are used for angiogenic evaluation of FTY720 delivery *in vitro* (Kocherova et al., 2019). Li et al., 2019 delivered FTY720 using a PLGA scaffold *in vitro* and *in vivo* (Li et al., 2019) and found that the FTY720 scaffold induced increased tube-like structures in HUVECs with longer tube lengths and higher number of branches. Similarly, Yang et al., 2022 showed similar results where HUVECs dispersed within an FTY720 loaded scaffold induced more tubule formation (Yang et al., 2022).

The physical characteristics of blood vessels induced by FTY720 delivery was studied to compare the maturity, the blood vessel investment, and arteriolar diameter. The maturity of blood vessels was analyzed by Das et al., 2014a, and they found that the number of mature vessels is substantially higher after treatment with FTY720 in a cranial defect model (Das et al., 2014a). Additionally, Li et al., 2019 stated that there was a 10% higher induced blood vessel volume when compared to non-treatment groups, which demonstrated the pro-regenerative effect that FTY720 has on angiogenesis (Li et al., 2019).

## 7 Conclusion

This review presents an in-depth analysis on the potential applications of FTY720 on regenerative wound healing through cell proliferation, immune cell modulation, collagen deposition and vascularization. Although FTY720 is an FDA approved treatment for multiple sclerosis, it is believed that FTY720 can have a pro-regenerative effect on wound healing via the delivery of a variety of biomaterials through the modulation of lymphocyte function. This review focused on results gained from *in vivo* studies delivering FTY720 into the injury models via biomaterials such as hydrogels, scaffolds, and subcutaneous catheterization. Though the epithelial, skeletal, and muscular wound healing models used different criteria for analysis, it is clear that FTY720 recruits pro-regenerative immune cells and increases vascularization, osteogenesis and collagen deposition through the expression of chemotactic signals (VEGF, TNF-α, IL-8). Variables yet to be considered in topical FTY720 delivery include systemic effects and concern for increased risk of infection. Although FTY720 is approved for human use in multiple sclerosis relapse patients, large animal studies to test FTY720 is the next step in testing FTY720 for wound healing in humans as an immuno-regenerative therapy.
